# Cloning and expression of human vascular endothelial growth factor gene and inhibition of its expression by antisense in prokaryotic system

**Published:** 2010

**Authors:** M. Yazdanfar, M. Bandehpour, F. Yarian, A Koochaki, K. Parivar, B Kazemi

**Affiliations:** 1 Islamic Azad University, Research and Sciences Campus; 2 Cellular and Molecular Biology Research Center, Shahid Beheshti University of Medical Sciences, Tehran. Iran

**Keywords:** Angiogenesis, Recombinant plasmid, Antisense transcript, Vascular Endothelial Growth Factor (VEGF)

## Abstract

**Background and the purpose of the study:**

Angiogenesis is an important process in physiology and disease pathogenesis and is controlled in a healthy body by a number of stimulatory and inhibitory factors. The aim of this study was to determine the effect of antisense transcript on the sense transcript of the endothelial growth factor (EGF) gene in bacterial system as an approach for the gene regulation in tumors.

**Methods:**

The hepatoma cell line (HepG2) was stimulated by PMA. VEGF mRNA was used for RT-PCR. VEGF cDNA was synthesised and cloned into T-vector pTZ57R, then sense fragment of VEGF subcloned into pACYC Duet-1 expression vector and antisense VEGF subcloned into pCDNA3 expression vector. Recombinant plasmids were transforemed into BL21 bacterial cells. Expression of recombinant plasmid was analysed by western blot technique.

**Results:**

The recombinant pCDNA3-VEGF (pYZantiVEGF) was successfully expressed in BL21 cells. Western blot analysis showed that the expression of VEGF decreased significantly in the cells transfected with VEGF antisense RNA compared with the pACYCDUET-1-VEGF (pYZsenseVEGF) transfected and control.

**Major conclusions:**

The expression of VEGF in BL21 cells was strong. In vitro, antisense of VEGF inhibited VEGF expression significantly in BL21 cells.

## INTRODUCTION

Angiogenesis is the process of primary vascular plexus formation, involving the differentiation of endothelial cells from in situ mesoderm-derived precursor cells via sprouting or non-sprouting process (1).

Angiogenesis occurs physiologically during embryogenesis and early post-natal development, and also during the process of wound healing and the reproductive cycle in adults (2). It is a complex process that is tightly regulated by stimulatory and inhibitory factors and initiated when there is a predominance of angiogenic factors that favour new vessel growth (e.g. Vascular Endothelial Growth Factor (VEGF), Fibroblast Growth Factor (FGF), and transforming growth factors alpha and beta), are commonly known as the ‘angiogenic switch’(2). VEGF is one of the most potent regulators of angiogenesis, both physiologically and pathophysiologically (2). It is a potent mitogen, a survival factor for endothelial cells, and also mediates vessel permeability and migration of endothelial progenitor cells from the bone marrow. As VEGF is secreted by tumors, it has limited role in adults, and is a rational therapeutic target (2). VEGF promotes the growth of tumour vasculature to allow oxygen and nutrients to reach the rapidly dividing cancer cells. However, this tumour vasculature is abnormal both in structure and function, with the vessels being immature, leaky and tortuous, with a reduced or absence of supporting cells (2, 3). The VEGF family consists of six different homologous factors, VEGF-A, placental growth factor (PlGF), VEGF-B, VEGF-C, VEGF-D, and VEGF-E. Although VEGF-A, PlGF, VEFG-B, VEGF-D and VEGF-E are important for the growth of blood vessels and VEGF-C mainly affects the development of lymphatic vessels. Recent evidences suggest that VEGF-A, VEGF-B and VEGF-C directly affect neural cells (4). The two main classes of receptors for the VEGF family are the tyrosine kinase and the non-tyrosine kinase receptors. The former contains three structurally related receptors: VEGFR-1 (Flt-1), VEGFR-2 (KDR/Flk-1), and VEGFR-3 (Flt-4). The non-tyrosine receptors consist of neuropilin-1 (NP-1) and neuropilin-2 (NP-2). The members of the VEGF family display different binding patterns to these receptors (5).

During the 1970s, the development of anti-angiogenic agents was proposed as an effective anticancer strategy (2). Evidence shows that anti-VEGF agents possess a variety of in vivo mechanisms of action comprising early effects of the regression of existing tumor microvasculature and normalization of remaining tumor vasculature, as well as continued effects such as inhibition of new tumor vasculature. The mechanism of action of the anti-VEGF agents with regards to direct antitumor effects has not been fully elucidated, and it is only suggested that inhibition of the effects of VEGF on dendritic cell maturation may improve the immune response to tumors (2). Gene therapy through gene transfer might be a promising way. Antisense gene technologies have proved to be powerful tools for selective regulation of gene expression in experimental settings and are under evaluation for their therapeutic potentials in clinic (6). Antisense agents downregulate the expression of specific target genes at mRNA level by pairing with their complementary RNA and preventing their translations into proteins Theoretically, antisense molecules could be used to cure a variety of diseases, especially some cancers (6, 7).

Increased expression of VEGF by tumor-associated vasculature is a feature of many human and rodent tumors and correlated with tumor growth rate, microvessel density, proliferation, tumor metastatic potential and poorer patient prognosis in many malignancies (8). Growth inhibition of human tumor cell lines (sarcomas, carcinomas and gliomas by anti-human VEGF monoclonal antibodies generated in mice correlates with the almost complete suppression of tumor-associated angiogenesis (8). Several angiogenesis inhibitors are presently very hot drugs in the clinical market place as anticancer agents. Antisense gene technologies have proved to be powerful tools for selective regulation of gene expression in experimental settings and are under evaluation for their therapeutic potentials in clinic (9). In this study effects of antisense transcription on the sense transcript of EGF gene of *E.coli* was investigated.

## MATERIALS AND METHODS

### 

#### Cells and plasmids

The HepG2 cell line (hepatocellular carcinoma) and pCDNA3 plasmid were gift from Dr.Naderian (Kashan University of Medical Sciences, Iran). The pACYCDuet-1 was purchased from Novagen (Germany).

#### Cell culture

The HepG2 cell line was maintained in RPMI (Roswell Park Memorial Institute, Sigma, Germany) containing 2 mM L-glutamine (Sigma, Germany), 100 U/ml penicillin (Sigma, Germany), and 100 µg/ml streptomycin (Sigma, Germany) supplemented with 10% fetal bovine serum (Gibco, UK). The plateau level for these cultures was about 106 viable cells/ml. The sufficient flasks were induced by 50 ng of phorbol myristate acetate (PMA) (Sigma, Germany)

#### RNA extraction and PCR amplification

VEGF RNA was extracted from HepG2 cell line. This gene was amplified via RT-PCR reaction. PCR product (1000 bp) was visualized in 1.5% agarose stained by syber green under ultraviolet light.

#### Gene cloning

Amplified fragment was cloned in pTZ57R plasmid by T/A cloning. The recombinant plasmid (pTZ57R/ VEGF) was transformed into *E. coli*, XL1 blue strain competent cell. The recombinant plasmid was extracted and digested by *Bam*HI and Hind*III* restriction enzymes.

The purified PCR product was cloned into *Bam*HI and Hind*III* digested pACYC DUET-1 and pCDNA3 expression vectors as sense and antisense plasmids respectively (pYZsensVEGF pYZantiVEGF). These vectors were transformed in *E. coli*, BL21. Recombinant plasmid was confirmed by both colony PCR and enzyme digestion methods. PCR reaction was carried out by specific primers for VEGF gene.

#### Expression of recombinant protein

The *E. coli* strain BL21 was transformed with the pACYC DUET-1/VEGF (pYZsensVEGF) and spread on Luria Bertani agar containing 50 µg/ml chloramphenicol. The transformant strain was inoculated into 3 ml culture tube containing modified YT medium (10) and allowed to grow at 37°C in a shaker at 160 rpm, overnight. The day after, it was sub-cultured into a 50 ml flasks containing YT medium and incubated at 37°C in a shaker, at 200 rpm. The culture in the logarithmic phase (at OD 600=0.6) was induced for 5 hrs and overnight with 1 mM IPTG as inducer. Samples were withdrawn and analyzed on 10% (v/v) SDS-PAGE, parallel with uninduced bacterial culture. The gel was stained with Coomassie brilliant blue R-250.

#### Transfer of sense and antisense plasmid in one bacterial host

Because two plasmids (pYZsensVEGF and pYZantiVEGF) contained two different origins of replication and two different antibiotic resistance genes, after pYZsensVEGF was transformed into BL21 bacterial cell, and used as host for antisense plasmid (pYZantiVEGF). Bacteria were resistant to two antibiotics which were used for screening. Gene expression was analyzed by 10% SDS-PAGE and western blotting.

#### Western blot analysis

Proteins resolved by SDS-PAGE were electro-phoretically transferred to a nitrocellulose membrane. The membranes was incubated in TBS (Tris-Buffered Saline containing 3% BSA (Bovine Serum Albumin) and then washed several times. The strips reacted with 1:200 dilution of anti VEGF monoclonal Ab (Anaspec.Cat.No.94555) and human serum respectively for one hour at 37°C.

The membrane was washed several times with TBS and TBS-T (TBS-Tween 20) and subsequently treated with horseradish peroxidase (HRP) conjugated sheep anti mouse Ig and rabbit anti human Ig at a 1: 100 dilution for one hour at 37°C. The strips were visualized for color after development in Di Amino Benzidine/H_2_O_2_ substrate solution for 15 min at The reaction was stopped by washing four times with distilled H_2_O

## RESULTS

**Figure 1:**
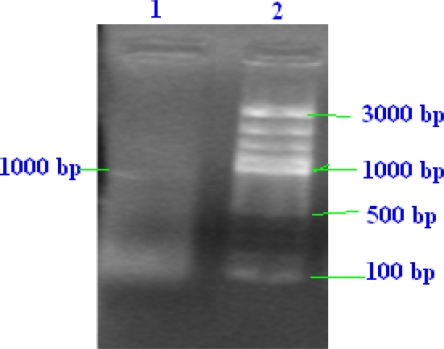
Electrophoresis of PCR on 1.5% agarose gels. Lane 1: The 1000 bp as PCR product of VEGF gene. Lane 2: GeneRuller 100-3000 bp DNA ladder marker

VEGF RNA was extracted and subjected to PCR amplification and PCR product was electrophoresed on 1.5% agarose gel (Fig 1).

**Figure 2:**
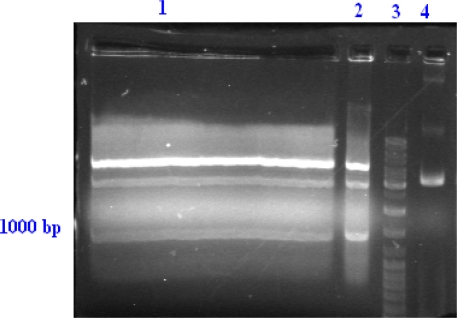
Electrophoresis on 1% LMP agarose gel Lane 1: Digested pTZ57R/VEGF by Bam*HI* & Hind*III* Lane 2: Digested pTZ57R/VEGF by Bam*HI* & Hind*III* Lane 3: GeneRuller 100-3000 bp DNA ladder marker Lane 4: not digested pTZ57R/VEGF

PCR product was ligated to pTZ57R/T vector and transformed into *E.coli* TOP10. White colonies containing recombinant plasmids were selected, extracted and electrophoresed on 0.8% agarose gel. For confirmation, the recombinant plasmid was digested (VEGF/ pTZ57R) by *Bam*HI and Hind*III* restriction enzymes, and released 1000 bp (VEGF gene). The digested recombinant plasmid is shown in figure 2.

Digestion reaction was electrophoresed on LMP agarose gel and released DNA band was sliced and purified by DNA extraction kit (Fermentas cat NO. k0513) and sub-cloned into *Bam*HI and Hind*III* digested pACYCDUET-1 expression vector and named pYZsensVEGF.

**Figure 3:**
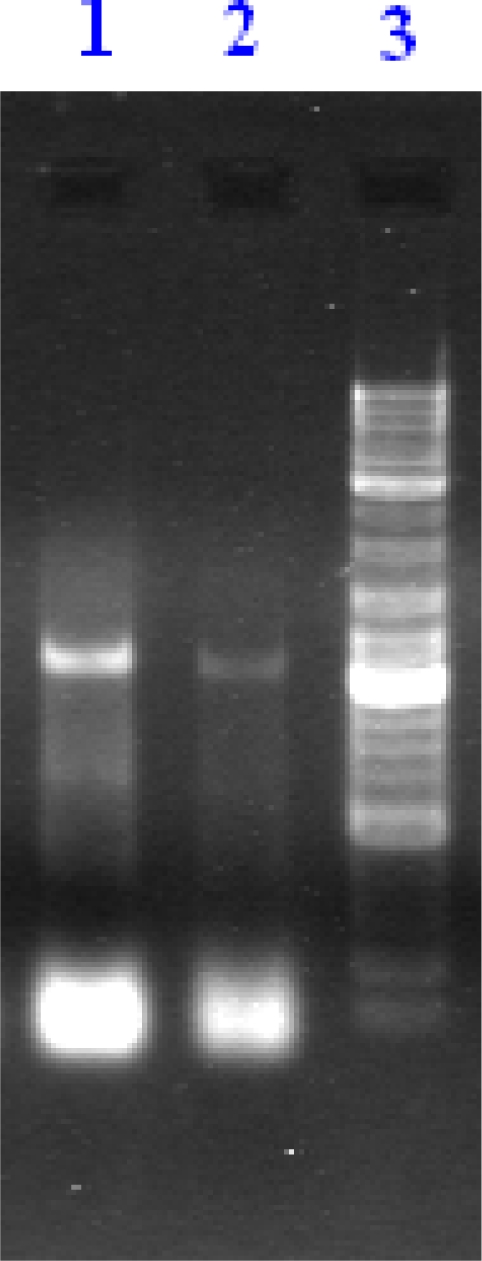
Orientation detection of cloned gene in expression Vector: 1.5% agarose gel electrophoresis of PCR product. Lane 1: 1000 bp PCR product as VEGF gene for confirmed subcloned gene in pACYCDUET-1 vector. (Using specific primer for VEGF) Lane 2: 1000 bp PCR product as VEGF gene for confirmed subcloned gene in pCDNA3 vector. (Using specific primer for VEGF) Lane 3: GeneRuller 100-3000 bp DNA ladder marker

For construction of VEGF antisense, Hind*III* and *Bam*HI digested fragment were ligated into pcDNA3 expression vector and named pYZantiVEGF. Subcloned gene in these vectors were confirmed using colony PCR, which was carried out by specific primers for VEGF gene (Fig 3).

### 

#### SDS-PAGE

**Figure 4:**
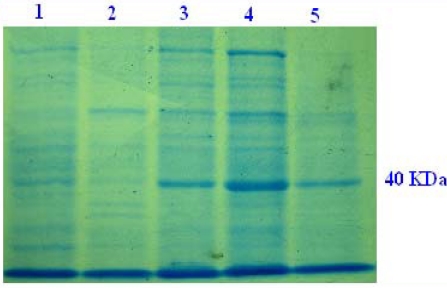
SDS-PAGE of bacterial lysate. Lane 1: Lysate of BL21 cell Lane 2: Lysate of BL21 cell containing intact pACYCDUET-1 Lane 3: Lysate of BL21 cell without induction containing pYZsensVEGF (0 h) Line 4: Lysate of BL21 containing pYZsensVEGF, collected 3hrs after induction Line 5: Lysate of BL21 containing pYZsensVEGF collected 3hrs after induction.

PYZsensVEGF was transformed into BL21 (DE3) *E. coli* and induced using 1 mM IPTG. Samples were taken before and at 3 hrs intervals after induction. The identity of the induced protein was confirmed by SDS-PAGE (Fig 4).

#### Western blotting

**Figure 5:**
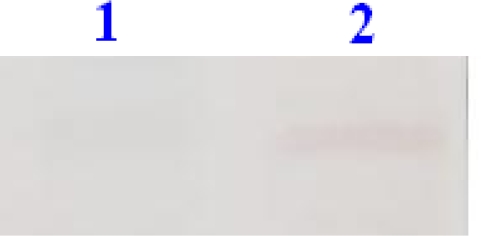
Immunoblotting. Lane 1: Cell lysates containing pYZsensVEGF and pYZantiVEGF 3hrs after induction (expression suppression) Lane 2: Cell lysates containing pYZsensVEGF 3hr after induction (expression)

The pYZsensVEGF was expressed and confirmed by western blot analysis, and no protein could be detected in the BL21 bacterial cells transformed by using VEGFR-specific CD8 cytotoxic lymphocytes pYZsensVEGF and pYZantiVEGF (Fig 5).

## DISCUSSION

Angiogenesis is an important process in physiology and disease pathogenesis. Angiogenesis is controlledin a healthy body by a system of angiogenic growth factors and angiogenesis inhibitors. When angiogenic growth factors are predominantly expressed, blood vessel growth occurs and disease may result. Successful therapies that target growth factors, their receptors, or the cascade pathways that are activated by growth factor/receptor interactions have been developed. There is good evidence that angiogenesis plays an important role in a wide range of cutaneous maladies, and angiogenesis-targeting therapies are playing an increasing role in the management of dermatological diseases. Cutaneous angiogenesis offers unexciting new arena for targeted dermatological therapeutics room temperature. (11). Therapeutic approaches based on tumor angiogenesis have been divided into two major classes; (i) vasculostatic agents and (ii) vasculotoxins (11).

Tumor blood vessels are distinct from normal resting blood vessels, and this feature makes them good targets for cancer therapy. In order to block tumor growth and metastasis formation, a number of inhibitors targeting the tumor vasculature have been identified by in vitro and in vivo anti-angiogenesis studies (31,32).

Antiangiogenic therapy targeting VEGF has been proposed as a means of inhibiting VEGF-dependent tumor growth and metastasis (9).

There are three types of anti-mRNA strategies:

1) Antisense oligonucleotide 2) Ribozyme/DNA Enzyme 3) Small interfering RNA.

Antisense agents act at the mRNA level, preventing its translation into protein. Antisense-oligonucleotides (AS-ONs) pair with their complementary mRNA, whereas ribozymes and DNA enzymes are catalytically active on ONs by binding and cleaving their target RNA. In addition, RNA interference has been established as a third highly efficient method for suppression of gene expression in mammalian cells by the use of 21-23-mer small interfering RNA (siRNA) molecules (12).

Rosen *et al* have shown that SU668 has a wide spectrum of activity and inhibits the tyrosine kinase activity of PDGFR, FGFR1 and VEGFR2 (13). Schueneman and his collaborators have shown that SU011248 inhibits VEGFR1 and PDGFR equally (14). Hurwitz et al showed the efficiently of Bevacizumab, a humanized antibody designed to target VEGF (15). Posey et al. showed that IMC-1C11, a chimerical IgG1 antibody against VEGFR2, is able to block ligand-receptor binding and inhibit phosphorylation (16). Niederman suggested that as opposed to antibodies could well be an original approach (17).

In this study, it was aimed to suppress VEGF gene with the help of its antisense. The available drugs for angiogenesis suppression will apparently have effects on the produced protein while in this study the proposed method was to suppress protein expression at a transcriptional level. Compared to other available techniques for suppression of protein expression, the antisense technique is simpler and cheaper.

Antisense agents down-regulate the expression of specific target genes at mRNA level by pairing with their complementary RNA and preventing their translation into proteins. Theoretically, antisense molecules could be used to cure a variety of diseases, especially some cancers (9, 18, 19). VEGF plays an important role in the angiogenesis. The use of antisense VEGF RNA for inhibiting vessel formation might be a rational approach.

## CONCLUSION

It was shown that VEGF expression was inhibited by antisense molecule in mRNA transcription level in prokaryotic system.
